# Association between off-hour admission of critically ill children to intensive care units and mortality in a Japanese registry

**DOI:** 10.1038/s41598-021-94482-0

**Published:** 2021-07-22

**Authors:** Takahiro Kido, Masao Iwagami, Toshikazu Abe, Yuki Enomoto, Hidetoshi Takada, Nanako Tamiya

**Affiliations:** 1 Department of Pediatrics, University of Tsukuba Hospital, 2-1-1 Amakubo, Tsukuba, Ibaraki Japan; 2 Department of Health Services Research, Faculty of Medicine, University of Tsukuba, 1-1-1 Tennodai, Tsukuba, Ibaraki Japan; 3 Health Services Research and Development Center, University of Tsukuba, 1-1-1 Tennodai, Tsukuba, Ibaraki Japan; 4 Department of Emergency and Critical Care Medicine, Tsukuba Memorial Hospital, 1187-299 Kaname, Tsukuba, Ibaraki Japan; 5 Department of Critical Care and Emergency Medicine, Faculty of Medicine, University of Tsukuba, 1-1-1 Tennodai, Tsukuba, Ibaraki Japan; 6 Department of Child Health, Faculty of Medicine, University of Tsukuba, 1-1-1 Tennodai, Tsukuba, Ibaraki Japan

**Keywords:** Paediatrics, Health policy

## Abstract

Limited information exists regarding the effect of off-hour admission among critically ill children. To evaluate whether children admitted to intensive care units (ICUs) in off-hour have worse outcomes, we conducted a cohort study in 2013–2018 in a multicenter registry in Japan. Pediatric (age < 16 years) unplanned ICU admissions were divided into regular-hour (daytime on business days) or off-hour (others). Mortality and changes in the functional score at discharge from the unit were compared between the two groups. We established multivariate logistic regression models to examine the independent association between off-hour admission and outcomes. Due to the small number of outcomes, two different models were used. There were 2512 admissions, including 757 for regular-hour and 1745 for off-hour. Mortality rates were 2.4% (18/757) and 1.9% (34/1745) in regular-hour and off-hour admissions, respectively. There was no significant association between off-hour admission and mortality both in model 1 adjusting for age, sex, and Pediatric Index of Mortality 2 (adjusted odds ratio [aOR] 0.89, 95% confidence interval [CI] 0.46–1.72) and in model 2 adjusting for propensity score predicting off-hour admission (aOR 1.05, 95% CI 0.57–1.91). In addition, off-hour admission did not show an independent association with deterioration of functional score.

## Introduction

Urgent medical needs can occur any time of the week. However, the quality of medical care might alter to some extent during “off-hours” due to the limited staff and restricted availability of medical equipment^1^. There are several studies addressing the association between patient outcomes and off-hour admission to intensive care units (ICUs)^2,3^. A meta-analysis has indicated higher mortality among critically ill adult patients admitted to ICUs during weekends than those admitted during weekdays^2^.

Although a study has suggested better outcomes among children admitted to ICUs during weekdays, there is a need for more comprehensive evidence^3^. In Japan, off-hour admission has not been associated with increased mortality among adult patients with brain stroke, acute myocardial infarction, sepsis, and trauma^4–8^. In contrast, no such evidence has been found for pediatric emergency admission to ICUs.

This study assessed a large cohort of a multicentered registry in Japan to investigate the association between off-hour ICU admission of severely ill children and mortality and neurological outcomes.

## Results

### Study subjects and their backgrounds

**Figure 1:**
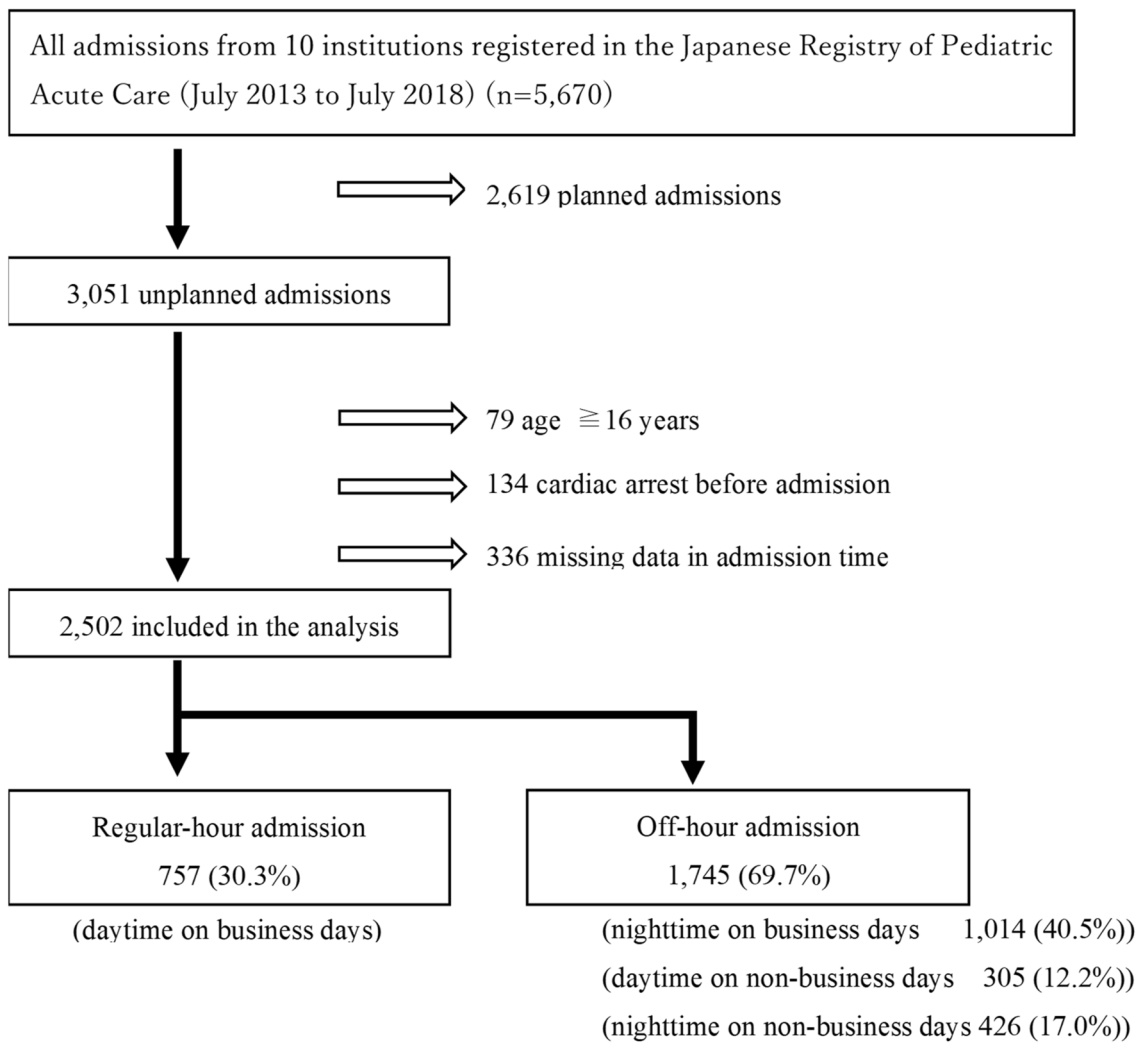
Flowchart of patient selection. Study subjects were selected from the data registered in the JaRPAC registry during the study period, according to the inclusion and exclusion criteria. Study subjects were grouped into regular-hours admissions (n = 757) and off-hours admissions (n = 1754). JaRPAC = Japanese Registry of Pediatric Acute Care.

A total of 5670 admissions were entered into the database during the study period between July 2013 and July 2018. Among them, 3051 (53.8%) unplanned ICU admissions were identified. After we applied the exclusion criteria, including adults (age ≥ 16 years), cardiac arrest before admission, and having missing data, 2502 admissions were eligible for the analysis. The final sample comprised 757 (30.3%) regular-hour and 1745 (69.7%) “off-hour” admissions (Fig. 1).

Table 1 shows the backgrounds of the patients included in each group. Patients admitted to the ICU in regular hours had a higher Pediatric Index of Mortality 2 (PIM2) compared with those admitted in “off-hours.” The place from where the patient was transferred and categories of primary diagnosis were different between the two groups. The “off-hour” group had a higher percentage of patients transferred from the emergency department (44.7% vs. 30.4%) and diagnosed with trauma (13.0% vs. 8.5%) at the time of admission than the regular-hour group.

**Table 1:** Baseline characteristics of the patients.

Variable	Regular-hour	Off-hour	*p* value
	(n = 757)	(n = 1745)	
Sex, men (%)	427 (56.4)	1011 (57.9)	0.477
Age (month), median (IQR)	26 (8–80)	28 (9–80)	0.35
PIM2, median (IQR)	1.5 (0.9–5.2)	1.2 (0.9–4.2)	< 0.001
The place from where the patients were transferred			< 0.001
ED (%)	230 (30.4)	780 (44.7)	
Transferred from other hospital (%)	361 (47.7)	659 (37.8)	
Operating room (%)	38 (5.0)	69 (4.0)	
Inpatient ward (%)	120 (15.9)	229 (13.1)	
Others (%)	8 (1.1)	8 (0.5)	
Primary diagnostic category			< 0.001
Respiratory (%)	260 (34.4)	574 (32.9)	
Neurological (%)	157 (20.7)	450 (25.8)	
Trauma (%)	64 (8.5)	227 (13.0)	
Gastrointestinal (%)	78 (10.3)	146 (8.4)	
Cardiac (%)	63 (8.3)	104 (6.0)	
Others (%)	135 (17.8)	244 (14.0)	

*PIM2* Pediatric Index of Mortality 2, *ED* emergency department in the same hospital, *IQR* interquartile range.

Comparisons between the groups were performed with the chi-squared test for categorical data and Mann–Whitney-U test for continuous data.

### Results of the main analysis for primary and secondary outcomes

The primary outcome was ICU mortality, and the secondary outcome was neurological sequelae, defined by any deterioration of the pediatric cerebral performance category (PCPC) at ICU discharge. The overall ICU mortality rates were 2.4% (18/757) and 1.9% (34/1745) in the regular-hour and “off-hour” admission groups, respectively. The proportions of patients with PCPC deterioration were 8.5% (64/757) and 6.9% (121/1745) in the regular-hour and “off-hour” admission groups, respectively (Table 2).

**Table 2:** Crude number and proportion of primary and secondary outcomes in each group.

Outcome	Regular-hour	Off-hour	*p* value
	(n = 757)	(n = 1745)	
**Primary outcome**			
Overall ICU mortality (%)	18 (2.4)	34 (1.9)	0.429
**Secondary outcome**			
PCPC deterioration (%)	64 (8.5)	121 (6.9)	0.106

*ICU* intensive care unit, *PCPC* pediatric cerebral performance category. PCPC deterioration was defined as any deterioration in PCPC score at discharge compared with PCPC score before admission.

The results of univariate logistic regression analysis and mixed-effects model multivariate logistic regression analysis (models 1 and 2) are summarized in Table 3. Univariate logistic regression analysis illustrated consistently low odds ratio (ORs) for primary and secondary outcomes in the “off-hour” group compared with those in the regular-hour group, although it was not significant (OR 0.79, 95% confidence interval [CI] 0.44–1.41 and OR 0.81, 95% CI 0.59–1.11, respectively).

**Table 3:** Results of univariate logistic regression and mixed-effects model multivariate logistic regression analysis.

Variable	OR	(95% CI)	*p* value	aOR*	(95% CI)	*p* value	aOR**	(95% CI)	*p* value
**Overall ICU mortality**									
Regular-hour	Reference			–			–		
Off-hour	0.79	(0.44–1.41)	0.430	0.89	(0.46–1.72)	0.738	1.05	(0.57–1.91)	0.880
**PCPC deterioration**									
Regular-hour	Reference			–			–		
Off-hour	0.81	(0.59–1.11)	0.183	0.90	(0.64–1.27)	0.560	0.90	(0.65–1.25)	0.530

*aOR* adjusted odds ratio, *ICU* intensive care unit, *PCPC* pediatric cerebral performance category, *CI* confidence interval.

aOR* was adjusted for age, sex, and PIM2 (model 1).

aOR** was adjusted for the propensity score calculated using patient background data (age, sex, PIM2 score, pre-admission PCPC, category of primary diagnosis, and source of admission) (model 2).

Two different multivariate regression models were employed in the current study, with model 1 adjusting for age, sex, and PIM2 and model 2 adjusting for propensity score predicting off-hour admission. The adjusted ORs (aORs) (95% CIs) for overall ICU mortality were 0.89 (0.46–1.72) and 1.05 (0.57–1.91) for models 1 and 2, respectively. Similarly, the aORs (95% CIs) calculated using multivariate models 1 and 2 for PCPC deterioration were 0.90 (0.64–1.27) and 0.90 (0.65–1.25), respectively.

### Results of sensitivity analysis

In sensitivity analysis, the association between “off-hour” admissions and early deaths (within-24-h death and within-48-h death) was also not significant (Supplementary Table S1). Subgroup analysis revealed no significant association between “off-hour” admission and outcomes in the non-surgical group. Both patient mortality and observed PCPC deterioration in the surgical group were significantly small to perform regression analysis (Supplementary Table S2). Among patients in the surgical group, the crude number and proportion of overall ICU mortality were 0/38 (0.0%) and 3/69 (4.4%) in regular-hour and “off-hour” admissions, respectively (Fisher’s exact, *p* = 0.551). None of the combinations of subgroups (business days vs. non-business days, or between nighttime admissions vs. daytime admissions) for ICU admission timing yielded significant differences in the primary and secondary outcomes (Supplementary Table S3). When patients were divided into four groups (daytime on business days, nighttime on business days, daytime on non-business days, and nighttime on non-business days) according to admission time, there was no significant difference in the proportion of outcome occurrence in each group (Supplementary Table S4).

## Discussion

To the best of our knowledge, this is the first study to investigate the association between “off-hour” admission and patient outcomes among critically ill children in Japanese ICUs. The results did not show a significant association between unplanned ICU admission in “off-hour” and mortality or deterioration of neuromotor status during ICU stay. The crude OR suggested a trend toward better outcomes in “off-hour” ICU admissions. However, the point estimate of aOR in both multivariate logistic models came close to 1, suggesting that this trend is probably due to the differences in background factors between the groups.

The crude overall ICU mortality was 2.3%, which was consistent with those of previous reports^9,10^ from other developed countries. Patient characteristics, such as age distribution and the prevalent disease categories (e.g., a higher number of trauma patients and less cardiac patients in the “off-hour” group), were also similar to those of previous studies, which examined the “off-hour” effect in pediatric ICUs (PICUs)^9^.

Past studies from other countries have not yielded consistent and robust evidence regarding the effect of “off-hour” admission among children admitted to ICUs. Most of them were conducted in single centers and did not consider the multiple covariates^11–15^. Only four studies have been conducted using multicenter registry analysis, making them sufficiently large to adjust covariates. Two studies conducted in the United States compared planned and unplanned admissions and suggested that “off-hour” admissions were associated with increased risk of mortality^9,10^. The other two studies dealt with unplanned ICU admissions alone, although their conclusions differed from each other^16,17^. Since the effect of “off-hour” admission might be influenced by the existing healthcare system, a large variation in outcomes might be observed across different regions. Therefore, there is a need to conduct such a study in each region^3^. Previously, Japanese researchers investigated the association between “off-hour” admission and patient outcomes for several critical diseases among adults and concluded no significant correlation^4–8^. Our study provided similar findings among children.

The reasons for the differences in the results of studies on “off-hours” care between Japan and other countries are multifaceted. This includes the varying staffing pattern of the ICUs in each country with different patient-to-nurse ratios globally. A distinctly high patient-to-nurse ratio of 2:1 is common in high-level ICUs and PICUs in many developed countries, including Japan^18,19^. Regarding physicians, there are no reports detailing the change in numbers between “off-hours” and regular hours. A common practice has been to have at least one person on duty at all times^20^. Considering the small number of beds per PICU in Japan (median 8)^21^ compared with that in Europe and the United States (median 12)^18^, the burden on physicians in charge of “off-hours” might be lesser in Japan. Furthermore, advanced practice providers such as nurse practitioners and respiratory therapists are not common in Japan. In contrast, in western countries, advanced practice providers play an important role in intensive care, although they may not be necessarily responsible for “off-hours.” This may be another reason for the difference in the burden on medical staff in charge of off-hours care between western countries and Japan^22^.

In contrast, some reports have suggested that the workload per staff member is rather small because the overall workload decreases during “off-hours”^23^. The average number of admissions for regular-hours and “off-hours” admissions using the current Japanese Registry of Pediatric Acute Care (JaRPAC) data suggests that there are 14 planned and 7 unplanned admissions per 100 h during regular hours and 2 planned and 6 unplanned admissions per 100 h during “off-hours.” In other words, unplanned admissions occur at a constant frequency regardless of time, whereas many planned admissions are added during regular hours. As a result, less than half as many admissions occur in “off-hours” as regular hours. There are many other factors to consider, such as operating room availability, severity of admitted patients, and prevalent disease groups. Large-scale, prospective studies with many explanatory factors are needed to consider a large number of covariates.

This study has some limitations. Due to its observational nature, we could not establish causality for exposure factors and outcome variables. Additionally, there might be unmeasured confounding factors such as the quality of procedures in emergency rooms and operating rooms. The characteristics of each facility, such as staffing and number of ICU beds, may be associated factors when considering the quality of off-hours care. However, the JaRPAC registry, by convention, does not allow for the identification of sites, the linking of site characteristics, or showing the results by site. Instead of adjusting for hospital-related factors, we accounted for the clustering effect of patients admitted in the same facility using a random-effects model.

In addition, the registry does not contain the socioeconomic status (SES) of the patients. From a global point of view, low SES is largely associated with a poor prognosis. However, the extent of such an association might be limited due to the provision of universal health care in Japan. There is a need to consider the association between SES and “off-hour” hospitalization. A report from South Korea, another country with a universal healthcare system, has found that low SES was not associated with a poorer prognosis among adult ICU patients^24^. Similarly, the influence of SES on “off-hour” hospitalization and eventual prognosis would also be expected to be minimal in Japan, although further study would be needed. Finally, we could not obtain data regarding process indicators (e.g., time to first antibiotics in sepsis and time to endotracheal intubation in airway obstruction), which might help to detect the causes of the outcomes observed. In addition, due to insufficient sample size, we were unable to perform a stratified analysis that would allow us to completely eliminate the effects of confounding factors, as shown in previous studies^11–15^. However, we adjusted confounding factors in two different models. We adjusted age, sex, and PIM2, which are considered fundamental factors for outcomes, and also adjusted for many confounding factors using a propensity score. None of the two adjusted or unadjusted results showed a significant difference. Finally, we should be aware that JaRPAC comprises volunteer institutions and might not be applied to other ICUs or PICUs. Furthermore, data entry errors might have occurred, possibly causing misclassification bias to dilute the association between the exposure and outcomes.

## Conclusions

We report the first multicenter cohort study in Japan, investigating the “off-hour” unplanned ICU admissions among children. No significant correlation was observed between “off-hour” ICU admissions and increased risk of mortality or poor neurological prognosis. This study could pave the way for future protocol development for the pediatric emergency care system.

## Methods

### Study design and setting

We conducted a cohort study using the JaRPAC^25^, an observational multicenter database. The establishment of the database was led by the pediatric committee of the Japanese Society for Emergency Medicine. The details of this registry are mentioned elsewhere^26^. Most of the large medical institutes and their associated hospitals in Japan have been included in the registry. The database includes 12 PICUs at children’s hospitals and 11 general ICUs at critical care centers, accounting for approximately 10% of the total ICU beds and 60% of the PICU beds in Japan^27^. The JaRPAC registry includes information on children admitted to ICUs (either pediatric or general ICUs). Since PICUs and children’s hospitals are not widely distributed throughout Japan, general ICUs cater to a large part of critically ill pediatric patients. All patients admitted to children’s hospitals are registered regardless of their age, whereas those admitted to other hospitals are registered if their age is < 16 years. No other exclusion criteria are applied to the registry. The database includes patient background data (age, sex, and past medical history), whether the admission was planned or not (an admission that could not be postponed for > 6 h was defined as unplanned admission), and the time of admission. It also includes the primary diagnosis and severity (defined by PIM2) at admission, preceding episodes of cardiopulmonary resuscitation, PCPC before admission to the ICUs, and ICU discharge status (death, PCPC, length of ICU stay, and destination). These are recorded by the physician in charge. The registry software program has been equipped with automated logic checks to avoid data deficiencies. Anonymized data can then be obtained by an investigator after approval of the proposed study protocol by the JaRPAC steering committee. Data on facilities are also anonymized, making it impossible to extract the characteristics of individual facilities. The publication of results for each facility is restricted.

### Ethical approval

This study was conducted according to the principles of the Declaration of Helsinki (2013). The study was approved by the Institutional Review Board (IRB) of the University of Tsukuba Hospital (H28-082). The IRB of the University of Tsukuba Hospital waived off the requirement for patients’ informed consent owing to the anonymized nature of the data is mentioned.

### Study subjects

All unplanned ICU admissions of children (age < 16 years) recorded in the JaRPAC database between July 2013 and June 2018 were included in this study. Owing to insufficient or incomplete patient information on admission or discharge, 336 patients (5.9% of total) were excluded from the study. Patients with a history of preceding cardiopulmonary resuscitation were also excluded because of confounding factors (pre-ICU admission status and quality of the resuscitation) altering the expected prognosis.

### Definition of exposure

This study aimed to assess “exposure” in terms of the timing of ICU admission. The doctors in charge of data entry into JaRPAC were instructed to select from the following four categories: daytime on business days, nighttime on business days, daytime on non-business days, and nighttime on non-business days. For analysis, we considered daytime on business days as “regular-hour” and the other three categories as “off-hour.”

### Outcomes

The primary outcome was overall ICU mortality. The secondary outcome was the deterioration of the PCPC. The PCPC is a widely used validated scale for neurological performance among children admitted to ICUs^28^. The PCPC comprises the following six categories: 1, normal; 2, mild disability; 3, moderate disability; 4, severe disability; 5, coma or vegetative; and 6, death. Deterioration of PCPC was defined as the increase in PCPC score during the period from pre-admission to discharge.

### Covariates

Demographics of patients (age, sex, place from where the patient was transferred, and pre-admission PCPC) and details of their disease condition (primary diagnosis and severity) were adjusted in statistical analyses to avoid confounding. PIM2^29^, a widely used severity index for PICU patients, was calculated and entered using software using patient background data and vital statistics.

### Statistical analyses

Descriptive statistics were assessed for demographic details and particulars of the disease condition. Continuous variables are expressed in medians and interquartile ranges, and categorical variables are expressed in numbers and percentages. The difference between groups was tested using the chi-squared test, Fisher exact test, or Mann–Whitney U test as appropriate.

Multivariate logistic regression analysis was separately performed for both primary (i.e., overall ICU mortality) and secondary outcomes (PCPC deterioration). A mixed-effects model multivariate logistic regression analysis, with hospitals as random effects and age, sex, and PIM2 as fixed effects, was performed (model 1). It was noted that other parameters (pre-admission PCPC, category of primary diagnosis, and place from where the patients were transferred) could still be important confounders affecting the outcomes. However, the small number of outcomes prevented us from adjusting for many confounding factors^14–18^. Thus, we summarized the aforementioned confounding factors into a propensity score and used it for statistical adjustment. The propensity score for “off-hour” admissions was calculated using a multivariate logistic model including several variables (i.e., age, sex, PIM2, pre-admission PCPC, category of primary diagnosis, and place from where the patient was transferred).

The aOR for primary and secondary outcomes among “off-hour” admissions was also calculated using a mixed-effects logistic regression model considering hospitals as random effects and the propensity score as fixed effects (model 2).

Several sensitivity analyses were performed. We analyzed exposure factors assuming within-24-h death and within-48-h death as outcomes, separately. This was to estimate the potential association between early death among critically ill patients and ICU admission timing. Furthermore, the main analysis was repeated with a division of the study population into two subgroups: surgical (those admitted from the operating room) and non-surgical. The main analysis was also repeated by differently grouping the timing of ICU admission (i.e., daytime on business days, nighttime on business days, daytime on non-business days, and nighttime on non-business days): business days vs. non-business days and daytime vs. nighttime as exposures. The propensity scores used in this analysis were recalculated with a logistic model that predicted non-business days and nighttime admissions, respectively. Finally, we divided the exposure into four time periods (daytime on business days, nighttime on business days, daytime on non-business days, and nighttime on non-business days) and compared the proportion of outcomes occurring in each group.

In our post-hoc sample size calculation using the Fleiss formula^30^ (alpha=0.05, power=80%, current sample size n=1745 in the “off-hour” group, n=757 in the regular-hour group) and proportion of primary outcome in the regular hour group (2.4%, as shown in the result section), it was projected that a statistically significant OR (“off-hour” vs. regular-hour) of ≥2.0 or ≤0.38 could be identified.

All tests were two-tailed, and a *p* value <.05 was considered statistically significant. Epi Info^TM^ version 7.2 (Centers for Disease Control and Prevention, USA) was used for sample size calculation. Statistical analyses were performed using STATA version 15 (StataCorp, Texas, USA).

## Supplementary Information

Supplementary Information.

## Data Availability

The data that support the findings of this study are available from the JaRPAC steering committee, although restrictions apply to the availability of these data, which were used under license for the current study, and so are not publicly available. Data are, however, available from the authors upon reasonable request and with permission of the JaRPAC steering committee.
