# Supplementary material for: Association between off-hour admission of critically ill children to intensive care units and mortality in a Japanese registry
Source: Sci Rep. 2021 Jul 22;11:14988. doi: 10.1038/s41598-021-94482-0 (PMC8298565; doi:10.1038/s41598-021-94482-0)
Supplement: Supplementary file 1 — Supplementary Information. [file 41598_2021_94482_MOESM1_ESM.docx]

| Supplementary Table S1. Results of additional analysis redefining outcomes as within-24-h death and within-48-h death | | | | | | | | | | | | | |
| --- | --- | --- | --- | --- | --- | --- | --- | --- | --- | --- | --- | --- | --- |
|  | | | | | | | | | | | | | |
| Variable | Incidence (%) |  | OR | (95% CI) | p value |  | aOR^*^ | (95% CI) | p value |  | aOR^**^ | (95% CI) | p value |
| Within-24-h death |  |  |  |  |  |  |  |  |  |  |  |  |  |
| Regular hour | 3/757 (0.4) |  | Reference |  |  |  | - |  |  |  | - |  |  |
| Off-hour | 5/1,745 (0.3) |  | 0.72 | (0.17–3.03) | 0.656 |  | 0.89 | (0.19–4.19) | 0.886 |  | 1.07 | (0.25–4.60) | 0.932 |
| Within-48-h death |  |  |  |  |  |  |  |  |  |  |  |  |  |
| Regular hour | 5/757 (0.7) |  | Reference |  |  |  | - |  |  |  | - |  |  |
| Off-hour | 7/1,745 (0.4) |  | 0.61 | (0.19–1.92) | 0.393 |  | 0.75 | (0.22–2.61) | 0.656 |  | 0.79 | (0.24–2.54) | 0.690 |

aOR adjusted odds ratio; CI confidence interval.
aOR^*^ was adjusted for age, sex, and PIM2 (model1).
aOR^**^ was adjusted for the propensity score calculated using patient background data (age, sex, PIM2 score, pre-admission PCPC, category of primary diagnosis, and source of admission) (model 2).

| Supplementary Table S2. Subgroup analysis for overall ICU mortality and PCPC deterioration in the surgical and non-surgical groups | | | | | | | | | | | | | | |  |
| --- | --- | --- | --- | --- | --- | --- | --- | --- | --- | --- | --- | --- | --- | --- | --- |
| **Surgical group** |  |  |  |  |  |  |  |  |  |  |  |  |  |  |  |
| Variable | Incidence (%) |  | OR | (95% CI) | p value |  | aOR^*^ | (95% CI) | p value |  | aOR^**^ | (95% CI) | p value | |  |
| Overall ICU mortality |  |  |  |  |  |  |  |  |  |  |  |  |  | |  |
| Regular hour | 0/38 (0.0) |  | Reference |  |  |  | - |  |  |  | - |  |  | |  |
| Off-hour | 3/69 (4.4) |  | Omitted |  |  |  | - |  |  |  | - |  |  | |  |
| PCPC deterioration |  |  |  |  |  |  |  |  |  |  |  |  |  | |  |
| Regular hour | 1/38 (2.6) |  | Reference |  |  |  | - |  |  |  | - |  |  | |  |
| Off-hour | 5/69 (7.3) |  | Omitted |  |  |  | - |  |  |  | - |  |  | |  |
| **Non-surgical group** | |  |  |  |  |  |  |  |  |  |  |  |  | |  |
|  | Incidence (%) |  | OR | (95% CI) | p value |  | aOR^*^ | (95% CI) | p value |  | aOR^**^ | (95% CI) | p value | |  |
| Overall ICU mortality |  |  |  |  |  |  |  |  |  |  |  |  |  | |  |
| Regular hour | 18/719 (2.5) |  | Reference |  |  |  | - |  |  |  | - |  |  | |  |
| Off-hour | 30/1,676 (1.8) |  | 0.71 | (0.39–1.28) | 0.256 |  | 0.79 | (0.40–1.54) | 0.489 |  | 0.92 | (0.50–1.70) | 0.805 | |  |
| PCPC deterioration |  |  |  |  |  |  |  |  |  |  |  |  |  | |  |
| Regular hour | 63/719 (8.8) |  | Reference |  |  |  | - |  |  |  | - |  |  | |  |
| Off-hour | 116/1,672 (6.9) |  | 0.77 | (0.56–1.07) | 0.117 |  | 0.87 | (0.61–1.23) | 0.427 |  | 0.87 | (0.63–1.21) | 0.410 | |  |
| aOR adjusted odds ratio; ICU intensive care unit; PCPC pediatric cerebral performance category; CI confidence interval. aOR^*^ was adjusted for age, sex, and PIM2. aOR^**^ was adjusted for the propensity score calculated using patient background data (age, sex, PIM2 score, pre-admission PCPC, category of primary diagnosis, and place from where the patient was transferred). | | | | | | | | | | | | | | |  |
|  |  |  |  |  |  |  |  |  |  |  |  |  |  |  |  |
|  |  |  |  |  |  |  |  |  |  |  |  |  |  |  |  |

| Supplementary Table S3. Results of analyses redefining exposures as non-business days and nighttime admission | | | | | | | | | | | |  |
| --- | --- | --- | --- | --- | --- | --- | --- | --- | --- | --- | --- | --- |
| **Non-business days (compared with business days)** | | | | | | | | | | | |  |
| Outcome | OR | (95% CI) | p value |  | aOR^*^ | (95% CI) | p value |  | aOR^**^ | (95% CI) | p value |  |
| Overall ICU mortality | 1.11 | (0.61–2.02) | 0.732 |  | 1.37 | (0.70–2.67) | 0.352 |  | 1.45 | (0.78–2.68) | 0.239 |  |
| PCPC deterioration | 0.97 | (0.70–1.35) | 0.860 |  | 1.02 | (0.72–1.46) | 0.903 |  | 1.09 | (0.77–1.53) | 0.633 |  |
| **Nighttime (compared with daytime)** | | | | | | | | | | | |  |
| Outcome | OR | (95% CI) | p value |  | aOR^*^ | (95% CI) | p value |  | aOR^**^ | (95% CI) | p value |  |
| Overall ICU mortality | 0.83 | (0.47–1.44) | 0.501 |  | 0.87 | (0.47–1.63) | 0.671 |  | 0.98 | (0.56–1.72) | 0.943 |  |
| PCPC deterioration | 0.82 | (0.61–1.11) | 0.191 |  | 0.91 | (0.66–1.27) | 0.611 |  | 0.91 | (0.67–1.24) | 0.550 |  |
| aOR adjusted odds ratio; ICU intensive care unit; PCPC pediatric cerebral performance category; CI confidence interval. aOR^*^ was adjusted for age, sex, and PIM2. aOR^**^ was adjusted for the propensity score calculated using patient background data (age, sex, PIM2 score, pre-admission PCPC, category of primary diagnosis, and place from where the patient was transferred). | | | | | | | | | | | |  |
|  |  |  |  |  |  |  |  |  |  |  |  |  |
|  |  |  |  |  |  |  |  |  |  |  |  |  |
|  |  |  |  |  |  |  |  |  |  |  |  |  |

| Supplementary Table 4. Crude number and proportion of primary and secondary outcomes of the four groups according to the timing of admission | | | | | |
| --- | --- | --- | --- | --- | --- |
|  | Daytime on  bussiness days | Nighttime on  business days | Daytime on  non-business days | Nighttime on  non-business days |  |
| Outcome | (n=757) | (n=1,014) | (n=305) | (n=426) | p value |
| Primary outcome |  |  |  |  |  |
| overall ICU mortality (%) | 18 (2.4) | 17 (1.7) | 6 (2.0) | 10 (2.4) | 0.726 |
| Secondary outcome |  |  |  |  |  |
| PCPC deterioration (%) | 64 (8.5) | 68 (6.7) | 23 (7.5) | 30 (7.0) | 0.566 |
| ICU intensive care unit; PCPC pediatric cerebral performance category. PCPC deterioration was defined as any deterioration in PCPC score at discharge compared with PCPC score before admission | | | | | |
|  |  |  |  |  |  |
